# Anatomical changes correlated with chronic pain in forensic medicine

**DOI:** 10.1080/20961790.2017.1341364

**Published:** 2017-06-30

**Authors:** Henry J Carson

**Affiliations:** Linn County Medical Examiner's Office (retired), Cedar Rapids, IA, USA

**Keywords:** Forensic medicine, pain, chronic, depression, emphysema, adhesions, tissue

## Abstract

This study was performed to determine the relationships between chronic pain and anatomic changes that may occur in the body. Autopsies were performed on fatalities that required death investigation in Linn County, IA, or adjacent and nearby areas. Persons with chronic pain were older than the control population at the time of death. Diabetes, hypertension and depression were more common in persons with chronic pain. Certain causes of death may also have been related to chronic pain. The heart, lungs, liver, spleen and kidneys were significantly heavier in persons with chronic pain; emphysema and pleural and abdominal adhesions were more common in persons with chronic pain. There appear to have been diffuse changes in the body related to chronic pain. These changes may have been mediated by a number of systemic mechanisms that are involved with chronic pain, including cardiovascular activity, the immune system, the neuroendocrine system and others.

## Introduction

Pain is defined as unpleasant localized or generalized physical sensations of discomfort or distress as reported by a person clinically that is associated with tissue damage or that can be experienced as such [1]. Pain is considered to be a chronic condition when it persists for three months or more [1–6], and it affects or disables millions of Americans [1,7,8]. The number of persons affected by chronic pain is not certain, but estimates range from 20% to 30% or even more of the adult population, especially the elderly and women [1–3,9–11]. Chronic pain is of interest in forensic medicine because it can be an important variable in quality of life [2–4,9,12,13], and may be managed by medications that lead to mortality [12,14]. This study examines the relationships between chronic pain, clinical factors and anatomic changes that may concurrently occur in the body.

Much is known about the physiology of chronic pain. The physical and functional neuroanatomy of chronic pain is being elucidated [2,7,8,15–27]. The roles of endogenous opioids, cytokines, chemokines and neurotransmitters in the central nervous system are under investigation [8,16,20,21,23,24,27]. The immune system appears to be intricately involved in pain functions as well [2,20,21,23,25,26,28,29]. Correlations of chronic pain have been made with the cardiovascular system as well, although anatomic changes are not typically demonstrated. The effects of pain on other organ systems are not as well characterized. This study investigated whether there are possible observable anatomic changes that may be related to chronic pain and that may be found in the investigation of death and by autopsy. The investigation also endeavours to associate its findings with physiological mechanisms that have been associated with chronic pain in other research.

## Materials and methods

Medical examiner cases were performed by the author in Linn County, IA, or adjacent or nearby counties from 1997 to 2014. Adult autopsies were performed to determine the cause and manner of death for standard statutory reasons, such as accident, suicide, homicide, unexpected death in a person with no previous medical history and others. Subjects were excluded in cases of severe decomposition, fire or animal activity that compromised the integrity or mass of the body or organs. Heart weights in cases that had undergone open cardiac surgery were removed from some calculations because they were artefactually heavy due to scarring and adhesions. Likewise, lung weights in cases of pneumonia and drowning were excluded in some calculations due to their artefactually higher weights. Children were excluded from the data, not because they cannot experience chronic pain states [1,6], but because their numbers were too few to contribute systematically to the data, and would provide essentially outlier data. A few teenagers who had effectively attained adult habitus were included, however.

Demographic data were collected, including age, sex and race. Autopsy findings included height, weight, organ weights, and external or internal changes due to injury, disease, or medical and social history. Body mass index (BMI) was calculated by a standard formula [30]. Other findings at autopsy that were specific to organ systems were also recorded. In the brain, the presence of cerebral oedema, trauma or haemorrhage was noted. In the heart, assessments of hypertrophy, atherosclerotic cardiovascular disease (ASCVD), myocardial infarct and coronary artery bypass grafting (CABG) were noted. Cardiac hypertrophy was based on established values of heart weight by body weight; nonsurgical hearts in this study that were two standard deviations above the mean for body weight were counted as hypertrophied [31]. In the lungs, the presence of pneumonia, emphysema and pleural adhesions was recorded. The presence of ascites and abdominal adhesions was noted. The presence of the appendix was recorded. In the liver, fatty change, hepatitis or cirrhosis was noted. The presence of a gallbladder was recorded. Other multisystem findings such as cancer were collected.

Records of investigations were examined, such as medications or drugs found on scene, and social history such as use of tobacco, caffeine, alcohol, marijuana or recreational drugs. Other important scene or investigative findings were recorded, such as evidence of accidents, blunt force injury, hanging, use of handguns, sharp objects, fire, carbon monoxide sources, asphyxiation/drowning and contemporaneous medical conditions, such as chronic diseases or new complaints. Medical history was collected from the investigation or medical records. Chronic pain was determined based on medical records and investigative findings. Chronic pain was defined as unpleasant localized or generalized physical sensations of discomfort or distress as reported by the decedent that was associated with tissue damage or that could have been experienced as such that lasted for more than three months [1]. In general, the precise date of onset of chronic pain was not known, but in 10 cases the decedents’ complete medical records were available for review, and the duration of chronic pain was 3 months to 34 years (mean 13 years). However, in the other cases, it was clear that the pain state was chronic based on descriptions such as *long-standing, long-term, prolonged*, *many years’ history of*, etc.; or by association with other chronic conditions, such as arthritis, diabetes or cancer. Information was also sought from investigation and medical records regarding hypertension, diabetes, depression, old or recent surgery, bipolar affective disorder, schizophrenia, substance abuse, seizure disorder or specific diseases associated with chronic pain such as fibromyalgia, inflammatory bowel disease, cancer and many others. Autopsy conclusions regarding the cause of death, manner of death, and toxicological results and interpretations were recorded.

The data were analysed with MedCalc for Windows, version 12 (MedCalc Software, Ostend, Belgium). Age was used as the hazard in the Cox proportional hazards regression analysis to determine the role of biological factors such as gender, race, height, weight and BMI in survival, and to analyse the role of common clinical factors such as diabetes, hypertension, chronic pain, and narcotic and alcohol use on survival. Organ weights were then used as hazards in the Cox analysis to determine the effect of chronic pain, age, sex, race and weight on the organs. A Cox analysis was also used to assess the possible contribution of medications on organ weights. Two-tailed *T*-tests were used to compare height, weight, BMI and organ weights between the chronic pain group and controls. A Kruskal–Wallis test was used to compare the medians of the number of medications used between the groups. Categorical data between the groups were compared by χ^2^ 2 × 2 contingency tables using Fisher's exact test.

## Results

Based on medical records and scene investigations, 54 persons had been diagnosed with chronic pain ante-mortem, and 320 had no known chronic pain. The demographic data are summarized in Table 1. The types of chronic pain encountered are listed in Table 2. Systemic or widespread pain was the most common type, such as neuropathic pain, fibromyalgia, multiple sites of arthralgia or pain from disseminated carcinoma; musculoskeletal or site-specific pain followed, particularly chronic low back pain.

**Table 1.** Subject data (case number).

Subject	Parameter/classify	Chronic (*N* = 54)	Control (*N* = 320)	Significance
Age (years old)	Mean ± SD	50 ± 13	44 ± 18	*P* = 0.020 4
	Range	29–82	13–88	–
Sex	Men	34	224	–
	Women	20	96	–
Race	Caucasian	52	269	*P* = 0.018 3
	Other	2	51	–
Height (inches)	Mean ± SD	68 ± 4	69 ± 4	–
	Range	60–75	55–77	–
Weight (pounds)	Mean ± SD	194 ± 66	180 ± 48	–
	Range	101–422	55–375	–
Body mass index (BMI)	Mean ± SD	29.0 ± 9.0	26.9 ± 6.6	–
	Range	15.8–52.7	10.2–57.1	–
Social	Caffeine	14	63	–
	Tobacco	1	23	–
	Alcohol	9	96	–
	Marijuana	3	48	–
Medical	Diabetes	10	21	*P* = 0.006 2
	Hypertension	18	52	*P* = 0.004 0
Psychiatric	Depression	28	60	*P* < 0.000 1
	Bipolar affective disorder	3	8	–
	Schizophrenia	0	5	–
	Substance abuse	13	42	*P* = 0.005 2

–: *P* > 0.05.

**Table 2.** Sites of chronic pain (*N* = 54).

Sites	Number
Systemic	20
Back	14
Abdomen	5
Neuropathic	6
Other musculoskeletal	5
Chest	2
Headache	2
Total	54

Death from natural causes was significantly more common among persons with chronic pain (chronic pain *n* = 28, control *n* = 109; *P* = 0.014 6). Homicide was significantly less common among persons with chronic pain compared to the control group (chronic pain *n* = 0, control *n* = 32; *P* = 0.007 8). There was no difference between accidents, suicide and indeterminate manners of death.

The Cox analysis of survival showed no differences based on sex, race, height, weight and BMI. Based on clinical factors (*P* = 0.000 4), systemic hypertension was significantly correlated (*P* = 0.000 8) with early mortality, while chronic pain, narcotic use, depression and diabetes mellitus did not appear to contribute significantly as covariates to overall survival.

The leading cause of death overall, blunt force injury, was significantly less common (*P* = 0.001) among persons with chronic pain (*n* = 2) compared to controls (*n* = 77). Fatalities from toxicological causes were significantly more common (*P* = 0.000 2) among persons with chronic pain (*n* = 16) compared to controls (*n* = 31). Persons with chronic pain (*n* = 6) were more likely (*P* = 0.012 2) to die from pneumonia compared to controls (*n* = 9). Fatalities from hanging, liver disease, diabetic ketoacidosis, cerebrovascular accident, sharp force injury, carbon monoxide poisoning, gastrointestinal haemorrhage, asthma, aortic aneurysm, seizure, asphyxiation, carcinoma and chronic obstructive pulmonary disease were not different among the groups.

The Cox analysis using organ weights as the hazard is reported in Table 3. Chronic pain was identified as a significant covariate in heart weight, along with advanced age, male sex, non-white race and increased body weight. Chronic pain was also identified as a significant covariate in spleen weight, along with Caucasian race and increased body weight.

**Table 3.** Cox regression analysis, organ weights as hazard.

Organs	Age	Sex	Race	Body weight	Chronic pain	Total *P*-value
Brain	*P* = 0.002 5	*P* = 0.000 1	–	*P* = 0.004 4	–	*P* < 0.000 1
Heart	*P* < 0.000 1	*P* < 0.000 1	*P* = 0.014 8	*P* < 0.000 1	*P* = 0.001 0	*P* < 0.000 1
Lung, right	–	–	–	*P* = 0.024 9	–	*P* = 0.011 4
Lung, left	–	*P* = 0.008 5	–	–	–	*P* = 0.024 2
Liver	–	–	*P* = 0.016 0	*P* < 0.000 1	–	*P* < 0.000 1
Spleen	–	–	*P* = 0.000 2	*P* < 0.000 1	*P* = 0.030 7	*P* < 0.000 1
Kidneys	–	*P* = 0.001 0	–	*P* < 0.000 1	–	*P* < 0.000 1

–: *P* > 0.05.

Anatomic findings from autopsy are summarized in Table 4. All of the visceral organs were significantly heavier in the chronic pain group compared to the controls. The median use of different medications and drugs in the chronic pain group was significantly greater (*P* < 0.000 001) in the chronic pain group (median 1, range 0–10), compared to the control group (median 0, range 0–8). Types of medications are summarized in Table 5, and the effects of these medications on organ weights are reflected in Table 6. However, when tested in the Cox analysis using organ weight as the hazard along with the other demographic factors determined previously to affect those weights, narcotics were the only class of drug identified as a significant contributor to the weight of the right lung (*P* = 0.003 2), while narcotics and other medications did not appear to contribute significantly to a difference in organ weights.

**Table 4.** Anatomic findings.

Organs	Parameter/classify	Chronic (*N* = 54)	Control (*N* = 320)	Significance
Brain	Weight (grams)	1 340 ± 167	1 362 ± 156	–
	Cerebral oedema	11	61	–
Heart	All (non-surgical), weight (grams)	426 ± 87	389 ± 118	*P* = 0.032 4
	Normotension, weight (grams)	401 ± 81	366 ± 100	*P* = 0.046 4
	Hypertension, weight (grams)	476 ± 78	505 ± 135	–
	Cardiac hypertrophy	35	89	*P* < 0.000 1
	Atherosclerotic cardiovascular disease (ASCVD)	26	148	–
	Myocardial infarction (MI)	3	9	–
Pleural cavities	Adhesions	11	13	*P* = 0.000 1
Lung, right	All, weight (grams)	639 ± 193	569 ± 230	*P* = 0.036 3
	Pneumonia(−), weight (grams)	641 ± 201	555 ± 213	*P* = 0.012 1
	Pneumonia(+), weight (grams)	630 ± 156	848 ± 352	–
Lung, left	All, weight (grams)	557 ± 220	492 ± 197	*P* = 0.028 3
	Pneumonia(−), weight (grams)	568 ± 235	484 ± 189	*P* = 0.007 9
	Pneumonia(+), weight (grams)	501 ± 116	639 ± 283	–
	Pneumonia	9	14	*P* < 0.002 5
	Emphysema	21	82	*P* = 0.044 6
Abdomen	Adhesions	7	6	*P* = 0.000 7
Appendix	Present	35	250	*P* = 0.000 6
Liver	Weight (grams)	2 026 ± 568	1 769 ± 544	*P* = 0.001 9
	Weight range (grams)	1 200–3550	700–3910	–
	Steatosis	27	135	–
	Cirrhosis	4	14	–
	Hepatitis	13	62	–
Gallbladder	Present	38	283	*P* = 0.000 2
Spleen	Weight (grams)	245 ± 112	186 ± 107	*P* = 0.000 3
Kidney	All, weight (grams)	339 ± 78	310 ± 94	*P* = 0.037 3
	Normotension, weight (grams)	335 ± 76	301 ± 90	*P* = 0.033 9
	Hypertension, weight (grams)	346 ± 84	356 ± 99	–

--: *P* > 0.05.

**Table 5.** Medication and drug use (case number).

Medication and drug	Chronic (*N* = 54)	Control (*N* = 320)	Significance
Narcotic	23	10	*P* < 0.000 1
Other pain medication	12	8	*P* < 0.000 1
Benzodiazepine	11	18	*P* = 0.000 9
Antidepressant	19	28	*P* < 0.000 1
Hypertensive	4	8	–
Anticonvulsant	1	7	–
Stimulant	4	30	–

--: *P* > 0.05.

**Table 6.** Organ weights based on significant medications, all patients (grams).

	Narcotics			Other pain medications			Antidepressants			Benzodiazepines		
Organs	Absent	Present	Significance	Absent	Present	Significance	Absent	Present	Significance	Absent	Present	Significance
Brain	1 357	1 372	–	1 359	1 344	–	1 362	1 334	–	1 356	1 389	–
Heart	390	420	–	391	433	–	394	387	–	392	406	–
Right lung	552	746	*P* < 0.000 1	564	635	–	570	545	–	563	624	–
Left lung	486	609	*P* = 0.002	492	568	–	498	476	–	491	554	–
Liver	1 782	2 124	*P* = 0.000 9	1 790	2 120	*P =* 0.012	1 774	2 033	*P =* 0.002 8	1 794	1 959	–
Spleen	187	271	*P* < 0.000 1	192	240	–	189	234	*P* = 0.008 7	191	237	*P* = 0.032 6
Kidneys	312	336	–	312	360	*P* = 0.025	313	325	–	312	339	–

–: *P* > 0.05.

## Discussion

This study investigates whether a documented experience of ante-mortem chronic pain may have been related to anatomic changes in the body that could be observed at autopsy. The task can seem daunting, since chronic pain does not have a single clinical signature. It has a number of causes and presentations [1], although they share a common experience of persistent distress that impairs one's experience of life, activities of daily living, work and relationships. It is important to consider that chronic pain is a very diverse condition arising from many aetiologies, so to consider them as a single diagnosis would be inappropriate. However, any type of chronic pain might be worth evaluating in death investigation, and from a medical investigative perspective, determining ante-mortem chronic pain from a number of aetiologies turned out to be achievable, even though the number of cases reviewed in this study was ultimately small. Interviews with survivors and review of medical records appear to have provided sufficient documentation to establish identifiable conditions that correlated with chronic pain, such as protracted low back pain, irritable or inflammatory bowel syndromes and fibromyalgia, among many others.

Demographically, people with chronic pain appeared to live longer than the control population. While the study was blinded to the presence of pain in the persons studied, this finding is probably due to a bias from studying a medical examiner population. Nearly all persons who die in accidents are autopsied, and fatalities from accidents tend to be younger than the general population [32], skewing their ages towards the younger decades. The trend for persons with chronic pain in this study to die from natural causes may be a reflection of this bias. No difference in gender was noted in this study, although nationally, women tend to be afflicted with chronic pain more frequently in the general population [1]. Caucasians in this study appeared to be more prone to experience chronic pain than controls. This finding may be due to greater access to care for chronic pain by Caucasians, or to differences in reporting of pain based on race or culture [1,33,34].

This study furthermore appears to find that the body may undergo changes that may be correlated with chronic pain. While many organs had interacting covariates in their size at the time of death, the heart and spleen were specifically correlated with chronic pain at the time of death, and all of the other visceral organs in persons with chronic pain weighed more than controls as independent variables. Systemic changes may suggest systemic mechanisms that cause the visceral organs to enlarge, such as those mediated by the central ner- vous system [2,7,8,15,17,18,20–26], peripheral nervous system [23], neuroendocrine system, endogenous opioids and cytokines [2,20,21,23,25,26,28], the circulatory system including blood pressure [8,10], serum factors [21,35–39], and the immune system [2,20,21,23,25,26,28]. Under the direction of these interacting systems, chronic pain may induce stress that leads to reactive enlargement of the organs. For example, systemic mechanisms that could enlarge the organs may include fluid redistribution to the interstitial space due to endocrine stimulation; immune cells may evoke inflammatory reactions; the central nervous system may stimulate sympathetic reactions; and others.

Chronic pain is not the sole cause of organ enlargement in this study. The Cox analysis demonstrates that brain mass decreases as a function of age, while it increases in male sex and as a function of body weight; heart mass, however, increases with age, male sex, non-Caucasian race, body weight and chronic pain; the right lung increases as a function of body weight, while the left lung is heavier as a function of male sex; the liver increases in mass according to Caucasian race and body weight; the spleen is heavier based on Caucasian race, body weight and the presence of chronic pain; and kidney mass increases as a function of male sex and body weight. Thus, the weights of the heart and spleen could be due to mechanisms specific to the organ. For example, the heart may enlarge as a reaction to the stress of chronic pain mediated by the central nervous system and baroreception [10]. Systemic hypertension could be a factor of increased heart mass as well. When viewed as a function of several covariates, increased heart weight correlated significantly with chronic pain and other factors. The spleen also appeared to enlarge as a covariate of chronic pain and others, possibly highlighting the role of immune system activation in pain reactions over time. The other visceral organs appeared to be significantly larger in the chronic pain group irrespective of the other variables just listed. The other covariates appear to have been significant factors in other ways, however, for example, the significantly increased mass of the liver and spleen in association with Caucasian race and increased body mass.

Medications appear to be independently associated with increased organ weights for several significant classes of drugs. However, when tested by the Cox analysis along with the previously identified factors that were correlated with organ weight, only one class of medication, narcotics, appeared to contribute significantly to the weight of the right lung. The meaning of this finding in the lung is not clear, but the implication in general is that medications frequently found in persons with chronic pain do not significantly increase the weights of the organs beyond the existing clinical or demographic factors.

Other anatomic differences may also suggest a reaction to chronic pain. For example, the surgical absence of the gallbladders and appendixes in persons with chronic pain suggests that these persons may be more alert to the experience of pain in general, leading to surgical intervention more frequently when the subjects observed abdominal pain compared to the control population. The significant incidence of pneumonia in persons with chronic pain could be related to decreased mobility in persons who experience ongoing pain, or the higher incidence of emphysema that was found in the pain group. Adhesions were more common in the pleural and abdominal cavities of the pain group, suggesting systemic inflammation. Conversely, there was no increased incidence of cerebral oedema, atherosclerotic cardiovascular disease, myocardial infarction, steatosis cirrhosis or hepatitis.

Clinically, the concurrence of depression and chronic pain is well known [2–4,9,12]. The cause of death in persons with chronic pain was significantly more likely to have been due to toxicological causes compared to controls. The greater use of opioid, antidepressant and sedative medications by persons with chronic pain, as well as the increased incidence of substance abuse, might increase the risk of fatalities from drug interactions. Also access to these medications in a context of ongoing distress and increased clinical depression could be related to suicide in this group [12], although the incidence was not significantly increased in this study. Fatalities from accidental overdoses and drug interactions were significantly higher than the control population, however.

The anatomic changes with the corresponding clinical or demographic correlations found in this study suggest that differences in the body are possibly correlated with chronic pain in many ways, such as organ enlargement, pneumonia, depression and increased likelihood of abdominal surgery such as cholecystectomy and appendectomy. The causes of death appear to demonstrate increased risk of death from pneumonia or toxicological causes in persons with chronic pain. The mechanisms of these changes are complex, and may include multiple factors.

There are some significant limitations to this study. The persons included in this study are drawn from medical examiner cases. This population is inherently biased because they are prone to be investigated and autopsied due to unexpected, sudden or violent death. Another limitation is that lapses of data may have occurred in some cases, for example, in the cases of organ or tissue donation. Also, given the importance of neuropathological findings in chronic pain, systematic brain and spinal cord dissections might have been valuable, although these studies were rarely practical. Finally, as noted, chronic pain is very diverse, and the pathophysiology of the changes observed in this study is at present speculative.
